# Utilization of skilled birth attendant at birth and associated factors among women who gave birth in the last 24 months preceding the survey in Gura Dhamole Woreda, Bale zone, southeast Ethiopia

**DOI:** 10.1186/s12889-019-7818-6

**Published:** 2019-11-11

**Authors:** Gizachew Sime Ayele, Abulie Takele Melku, Semere Sileshi Belda

**Affiliations:** 1 Gura Dhamole Woreda, Rayitu Health Center, Primary Health Care Unit, Bale Robe, Ethiopia; 2 Department of Public Health, Madda Walabu University Goba Referral Hospital School of Health Science, Bale Goa, Ethiopia

**Keywords:** Skilled birth attendant, Obstetric danger signs, Utilization, Birth preparedness

## Abstract

**Background:**

Maternal morbidity and mortality continued to be major issues in many countries. Globally a total of 10.7 million women have died between 1990 and 2015 due to maternal causes where sub-Saharan Africa alone accounts for 66% of maternal death. Since most maternal deaths are avoidable; skilled attendance during pregnancy, childbirth, and the postpartum is among the most critical interventions for improving maternal and neonatal survival. The study aimed to assess the magnitude and associated factors of utilization of skilled birth attendant at birth among women who gave birth in the last 24 months preceding the study in Gura Dhamole Woreda, Bale Zone Southeast Ethiopia, 2017.

**Methods:**

Community based cross-sectional study was implemented from March 25 to April 24, 2017 in Gura Dhamole Woreda on total of 402 study subjects who were selected by Multi-stage sampling technique. The data were collected using pre-tested structured questionnaire and data was coded, entered, cleaned and analyzed using Statistical Package for Social Service (SPSS) Version 20. Odds ratio with 95% Confidence Interval (CI) was used to assess associations the dependent and independent variables. Logistic regression model was employed to identify independent predictors and variables were declared statistically significant at *P* value < 0.05.

**Result:**

In this study only 29.2% of women were assisted by Skilled Birth Attendance (SBA) during their child birth. Place of residence, mother education, travel time, joint decision on the place of delivery, ANC visit frequency, birth preparedness and complication readiness status, knowledge on obstetric danger signs after delivery and knowledge of presence of maternity waiting homes were significantly associated with SBA utilization.

**Conclusion:**

Skilled birth attendant utilization in the study area was low. Strategies that improve attendance of antenatal care utilization and attention to birth preparedness and complication readiness and counseling on danger signs are recommended.

## Background

Delivery attended by skilled professionals is known to contribute to the better outcome of pregnancy and child birth, for early detection and management of complications in the ANC period, during delivery and postnatal period. Therefore skilled attendant have positive contribution in the reduction of maternal and newborn mortality and morbidity [1].

Despite the existence of such means of reducing maternal mortality is a problem for many counties. Women in developed countries are still suffering from problems that occur at the time of pregnancy, labor, and delivery [2]. Pregnancy and childbirth are major problems of mothers in less developed and economically disadvantaged countries [3]..

Most maternal deaths are related to obstetric complications particularly in low income countries [3]. The five leading causes of maternal death in Ethiopia are unsafe abortion, sepsis (infection), hemorrhage, high blood pressure during pregnancy (preeclampsia and eclampsia) and obstructed labor [4, 5]. The consequences of maternal mortality and morbidity are felt not only by women but also by their families and communities. Children who lose their mothers are at an increased risk of death or other problems, such as malnutrition. Loss of women during their most productive years also means a loss of resources for the entire society [6].

Since all pregnancies can have a risk to the mother and lead to maternal death, ensuring skilled attendant is mandatory in order to prevent and mange complications by proven solution [4]. Hence, high-quality antenatal and intranatal care is critical to identify and safely manage complications. The skilled attendant is critical in this continuum of care, as a healthcare system that is appropriately prepared, supplied, staffed, and supported by emergency referral transport [7].

To improve utilization of health facilities with skilled birth attendant during delivery in the country, barriers to utilization of health facility with SBA during delivery need to be identified across all geographical regions. Little is known in the study area on current magnitude of utilization of maternity services in health facilities particularly skilled birth attendant services and determinants for their utilization.

Therefore, this study was aimed to assess the magnitude of skilled birth attendant utilization and identify factors associated with skilled birth attendant utilization among mothers who gave birth in the past 24 months in Gura Dhamole Woreda, Bale Zone, Oromiya Regional State, Southeast, Ethiopia. This information is important for informed decisions making by different stakeholders working to improve maternal and child health in pastoralist community in general and the study area in particular.

## Methodology

### Study area

The study was conducted Gura Dhamole woreda which is found 557 km away from Addis Ababa and 125 km East of Robe town, the Capital City of the Bale zone. Gura Dhamole is one of the pastoral woreda’s in Bale Zone and the woreda is bounded by Madda Walabu woreda in northeast, Barbare woreda and Somale regional state in East, Goro woreda in southwest and Dawe Kachan woreda in the southeast. The woreda have 2 urban and 15 rural kebeles. The total population of the woreda is estimated to be 37,918 according to 2016 population estimation based on Ethiopian national census of 2007. From this 8391 are women of childbearing age, 1316 are pregnant women. Regarding the health infrastructure, the woreda have 3 health centers and 14 health posts. Thirty skilled health professionals; 6 health officers, 9 diploma mid-wife nurses and 15 clinical nurses are working in the woreda [8].

### Study period

A community based cross-sectional study was conducted to assess the magnitude of skilled birth attendant utilization and to identify associated factors that were assumed to be barriers/facilitator to skilled birth attendant utilization among mothers who gave birth in the past 24 months in Gura Dhamole Woreda, Bale Zone, Oromiya Regional State, Southeast Ethiopia. The source population was all women who gave birth in the last 24 months prior to the survey in Gura Dhamole. Those women who gave birth in the last 24 months prior to the study irrespective of the outcome of the birth and place of delivery in six randomly selected kebeles were the study population. Those women who were permanent residents (those who lived more than 6 months) of randomly selected kebeles and sampled household and who gave birth from March 25, 2015 to April 24, 2017 were included in the study. Those women who were mentally and physically sick and unable to respond to interview and women who lived for less than 6 months in study area were excluded from the study.

### Sample size and sampling procedures

The sample size was determined using single population proportion formula, assuming SBA utilization of Oromiya Regional state of 19.7% [9], 95% level of confidence, 0.05 margin of error and 1.5 design effect was used since multi stage sampling technique was used and 10% was added non response rate and the final sample was 402 women who gave birth in the last 24 months.

Multistage sampling method was used to select respondents. First the study area was stratified into urban and rural kebeles. There were 15 rural and two urban kebeles in the woreda. From 2 urban and 15 rural kebeles, one urban and five rural kebeles were selected by lottery method.

House-to-house visit was carried out in selected kebeles to identify households with women who gave birth in the last 24 months prior to the survey and 904 households were identified fulfilling eligibility criteria. By allocating the sample size proportionally to each kebeles, using the list household as a sampling, systematic random sampling was used with a sampling interval of two. If the houses were closed or the mother was not present at the time of data collection, revisits were made until data collector got the women for the data collection. However, the next household was considered in case eligible women could not be accessed. When there were more than one eligible woman in the selected household, lottery method was used to select the one to be included in the study.

### Study variables

The dependent variable is utilization of skilled birth attendance. And independent variables were socio demographic characteristics (age and place of residence), socioeconomic factors (educational status of respondent and husband, occupation, family size, monthly income and communication media ownership), obstetric factors (age at first marriage, gravida, para, history of abortion and/ or still birth, pregnancy status, antenatal visit, counseling, place of delivery, birth preparedness, delivery attendant and duration of labor), husband’s factors (educational status and occupation), Knowledge of mothers on key danger signs of pregnancy/childbirth, infrastructure and health service related factors (availability, travel time, cost for service and ambulance service) and decision making on place of delivery. Utilization of skilled birth attendance was assessed by asking the mother if she gave the last birth at health center or hospital.

### Operational definitions

For the purpose of better understanding of readers the following terms and phrases were operationally defined for the current study.

**Skilled attendant**: - is a professionally trained health worker having the essential midwifery skills to manage normal labor and delivery, recognize complications early and perform any essential interventions including early referral.

**SBA utilization: -** Women who gave birth in health center and hospital by assistance of health professionals that have midwifery skills including Midwife nurse, Nurse, Health Officers and Doctors.

**Knowledgeable on advantage of pregnancy and delivery services:-** Women were considered knowledgeable if they mentioned advantages of pregnancy, delivery service and use SBA if they list two and above two advantages correctly from 4 lists and not knowledgeable if otherwise.

**Knowledgeable on danger signs of pregnancy: -** A woman was considered as knowledgeable if she can mention at least three key danger signs that could occur during pregnancy.

**Knowledgeable on danger signs of labor/childbirth: -** A woman was considered as knowledgeable if she can mention at least three danger signs that could occur during Labor/childbirth.

**Knowledgeable on key danger signs of postpartum: -** A woman was considered Knowledgeable if she can mention at least the three danger signs that could occur during postpartum period /after delivery.

### Data collection instrument and procedure

Data was collected through face-to-face interview using pre-tested structured questionnaire developed after reviewing relevant literatures [10–12] in English and translated to local language, Afan Oromo and back translated to English to check its consistency. The questionnaire had five parts; those were socio-demographic characteristics, obstetric history of respondents, knowledge and attitude of mothers on pregnancy and labor/childbirth, Reinforcing factors and enabling factors and maternity waiting area usage. Six data collectors who were outside of the study areas were recruited and trained on the face-to-face interview. Additionally the supervisor was trained on how to supervise the data collection process.

### Data quality control

To ensure data quality the questionnaire was translated into local language of study area i.e. Afan Oromo, for better understanding among data collectors and respondents. Pre-tested was done on 20 women at Funale Kosii kebele (outside of selected kebeles). Findings and experiences from the pre-test was used in modifying the questionnaire. Training was given to the data collectors and supervisors for two consecutive days before the actual data collection regarding the aim of the study, questionnaire and procedures of data collection.

The data collection process was supervised by the investigator and supervisor. The investigator and supervisor randomly revisited some of the households; checked if the eligible women were interviewed using systematic sampling technique from list of eligible women prepared during house-to-house visit. Data collectors were made to check questionnaire for completeness before leaving the participant and the supervisor and investigator reviewed each questionnaire on daily basis and checked for completeness. Those questionnaires found to be incomplete were completed by revisiting the household.

### Data analysis

The data was coded and entered into SPSS windows version 20.0 statistical software by investigator. Then, the entered data was cleaned for errors prior to data analysis. Frequencies were used to check for missed values and outliers during analysis. Any errors identified were corrected after revision of the original data using the code numbers given to each questionnaire. The descriptive analyses such as percentages, measures of central tendency were conducted. Statistical association between dependent variable and covariates was done using logistic regression. Significance was determined using crude and adjusted odds ratios with 95% confidence intervals. To assess the association between the different factors variables of skilled birth attendant utilization with the dependant variable (Use of SBA), first relationships between each independent variable and dependent variable was investigated using a binary logistic regression model. Those independent variables that have association with use of SBA at the bivariate level at *P* < 0.05 were included in a multivariate logistic regression model, after controlling the possible effects of confounders and variable that had significant association were identified by calculating odds ratio with 95% confidence interval and those independent variables with *P*- value < 0.05 were declared statistically significant.

### Ethical consideration

The proposal was approved by Madda Walabu University Goba Referral Hospital department graduate committee for ethics. An official letter was written from the Madda Walabu University Goba Referral hospital department of public health to Gura Dhamole woreda health and administrative office to seek cooperation.

During data collection respondents were informed about the objective of the study and procedure of selection as well as assured that their response will be kept confidential and their names were not registered; only code was used to identify the respondent’s response to minimize social desirability bias and assure confidentiality. Participation in the study was on voluntary basis and informed verbal consent was obtained from each study participants before actual data collection and participants were informed that they have the right to withdraw from the interview at any time without restriction of any of their benefits and this was approved by ethical review board of Madda Walabu University. In this study there are no minors aged less than 16 years participated.

## Results

From a total of 402 sampled mothers, 387 women who gave birth in the last 24 months were interviewed making a response rate of (96.3%) and 15 (3.7%) questionnaires were incomplete excluded from the analysis. The mean age of respondents was 26.4 (±5.87Standard Deviation (SD)) years. Three hundred twenty two (83.2%) of the respondents were rural inhabitants (Table 1).

**Table 1:** Socio-demographic and socio-economic characteristics of the respondents in Gura Dhamole woreda, Bale zone, Southeast Ethiopia, April, 2017

Variable	Response	Frequency	Percentage
Age	15–19	39	10.1
	20–24	117	30.2
	25–29	125	32.3
	30–34	52	13.4
	35–44	43	11.1
	40–44	11	2.8
Place of residence	Urban	65	16.8
	Rural	322	83.2
Educational status of Respondents	No formal Education	246	63.6
	Primary Education Secondary and above	10,833	27.98.5
Educational status of husband (*n* = 383)	No formal Education Primary Education	197	51.4
	Secondary and above	13,749	35.812.8
Occupational status of mother	House wife Farmer and Livestock rearing	257	66.4
	Government employed	91	23.5
	Merchant and Other^a^	1524	3.96.2
Occupational status of husband (*n* = 383**)**	Farmer	219	57.2
	Livestock rearing	112	29.2
	Government employed	25	6.5
	Merchant, Daily labourer and Other^a^	27	7
Family size	≤4	142	36.7
	5–6	110	28.4
	≥7	135	34.9
Monthly income (*n* = 386)	< 500	55	14.2
	500–1000	117	30.3
	≥1001	214	55.4
Means of communication	Radio	208	53.7
	TV /Telephone	23	6
	Mobile phone	168	43.4
	None	116	30

Other^a^ (student)

### Obstetric characteristics of respondents

In this study 268 (69.3%) of the respondents have got first marriage before the age of 18 years with mean age of first marriage 16.48 years (±2.01 SD). Three hundred twenty five (84%) of respondents have got their first pregnancy before the age of 20 years. Sixty six (17.1%) and 36 (9.1%) of the respondents had history of abortion and still birth respectively. One hundred twenty three 123 (31.8%) of women gave birth at health facilities for the last child birth. Majority of respondents, 264 (68.2%) delivered at home without assistance from skilled birth attendant (Table 2).

**Table 2:** Age at first marriage and pregnancy, BPACR, skill attendance utilization, counseling and duration of last labor in Gura Dhamole woreda, Bale zone, Southeast Ethiopia, April, 2017

Variables	Response	Frequency	Percentage (%)
Age at first marriage	< 18 yrs	268	69.3
Above	18 years and	119	30.7
Age at first pregnancy	< 20 yrs	325	84
	≥ 20 years	62	16
Gravidity	1	65	16.8
	2–4	151	39
	5 and above	171	44.2
Parity	1	67	17.3
	2–4	159	41.1
	5 and above	161	41.6
Ever had history of abortion	Yes	66	17.1
	No	321	82.9
Ever had history of still birth	Yes	36	9.3
	No	351	90.7
Last pregnancy planned	Yes	174	45
	No	213	55
ANC visit during last pregnancy	Yes	289	74.7
	No	98	25.3
ANC Frequency(*n* = 289)	< 4 visit	178	61.6
	4 and above	111	38.4
Counselled on Place of delivery during ANC visit(*n* = 289)	Yes	262	90.7
	No	27	9.3
Ever used HF for delivery service before last child birth(*n* = 320)	Yes	82	25.6
	No	238	74.4
BPACR (arrangement) taken	Identified place of delivery	223	57.6
	Identified place of delivery Identified skilled provider	116	30
	Saved money	212	54.8
	Identified means of emergency transport	37	9.6
	Arranged a blood donor for emergency	18	4.7
	Identified emergency signs	33	8.5
	Identified HI with 24 h EmOC	45	11.6
No of BPACR actions or arrangements taken	0	49	12.7
	1	118	30.5
	Well prepared(≥ 2 arrangements)	220	56.8
	Not well prepared(< 2 arrangement)	167	43.2
Place of delivery in the last 24 months	Health centre	101	26.1
	Hospital/ Health post	22	5.7
	Home	264	68.2
Assistance during health centre and hospital delivery	Mid wives	94	83.2
	Nurses /Health officers /Doctors	19	16.8
Duration of last labour	< 12 h	368	95.1
	≥12 h	19	4.9

Seventy six (19.6%) of the respondents had experienced adverse pregnancy and child birth outcome at their last child birth. The complications mentioned were vaginal bleeding 21 (27.6%), prolonged labor 31 (40.8%), retained placenta 34 (44.7%), increased blood pressure 10 (13.2%) and others 1(1.3%).

**Fig. 1:**
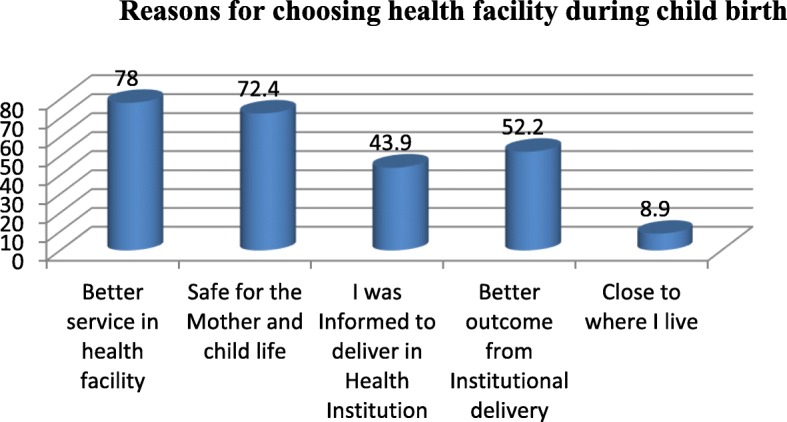
Reasons for choosing health facility during delivery among respondents in Gura Dhamole woreda, Bale zone, Southeast Ethiopia, April, 2017

**Fig. 2:**
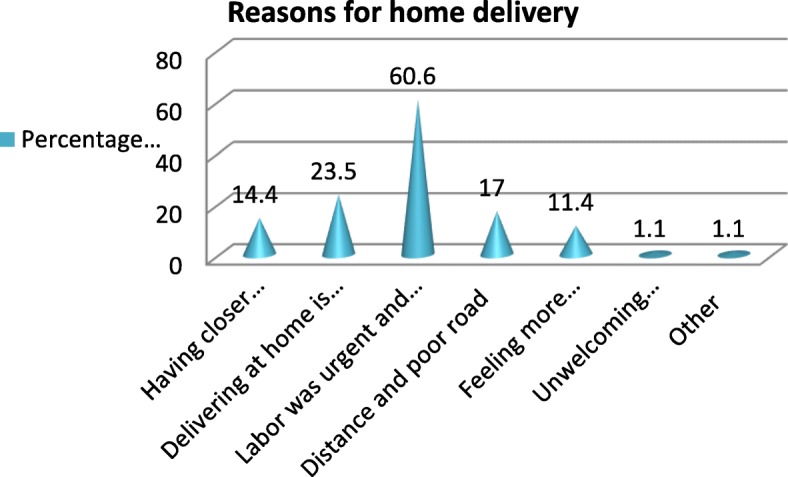
Reasons for home delivery among respondents in Gura Dhamole woreda, Bale zone, Southeast Ethiopia, April 2017

The reasons respondents gave for institutional delivery were better service in health facility 96 (78%) and safe for the mother and child life 89 (72.4%) (Fig. 1). Among women who delivered at home, 101 (38.3%) were assisted by Traditional Birth Attendants (TBA), no one or without any assistance 72 (27.3%), mother 52 (19.7%), family 49 (18.6) and others (neighbors) 5 (1.9%) (Fig. 2).

Regarding to preference for future place of delivery 316 (81.7%) of respondents preferred to deliver at health institution, 35 (9%) at home and 36 (9.3%) had no decision. The reasons for preferring institutional delivery respondents for future were had faced poor outcome from home delivery 210 (66.5%), newborn had faced poor outcome from home delivery 124 (39.2%), it was against their intention that they delivered at home 96 (30.4%), fear of Human Immune Virus (HIV) infection 41 (13%) and other reasons such as being delivered at health institution previously, to get improved and enough service and to get skilled birth attendant service 11 (3.5%).

### Knowledge and attitude about pregnancy, labor and delivery service

In this study women were considered knowledgeable on obstetric danger sign if they were mentioned at least three key danger signs. In this study only 105 (27.1%) women were knowledgeable on key danger signs of pregnancy, 130 (33.6%) and 149 (38.5%) women were knowledgeable on key danger signs during labor and child birth and post partum (after delivery) period respectively (Table 3).

**Table 3:** Knowledge status on key obstetric danger signs during pregnancy, labor, child birth and after delivery in Gura Dhamole woreda, Bale zone, Southeast Ethiopia, April, 2017

Variables	Response	Frequency (*n* = 387)	Percentage (%)
Knowledge on danger of obstetrics problems related to pregnancy	Not knowledgeable (knew < 3 key danger signs)	282	72.9
	Knowledgeable (Knew ≥3 key danger signs)	105	27.1
Knowledge on danger of obstetrics problems related to Labor and child birth	Not knowledgeable (knew < 3 key danger signs)	257	66.4
	Knowledgeable (Knew ≥3 key danger signs)	130	33.6
Knowledge on danger of obstetrics problems related to postpartum	Not knowledgeable (knew < 3 key danger signs) Knowledgeable (Knew ≥3 key	238	61.5
	danger signs)	149	38.5
Knowledge on Obstetric problems that can occur during pregnancy (*n* = 332)	Vaginal bleeding	168	50.6
	Severe headache	163	49
	Blurred vision	146	44
	Severe abdominal pain	129	38.9
	Loss of consciousness	76	22.9
	Convulsion	50	15.1
Knowledge on obstetric danger signs related to labour and child birth (*n* = 318)	Severe vaginal bleeding	199	62.6
	Severe headache	171	53.8
	Retained placenta	160	50.3
	Labor lasting > 12 h	124	39
	Loss of consciousness	91	28.6
	Others	2	0.6
Knowledge on danger signs after delivery/during post partum period (*n* = 353)	Retained placenta	240	67.8
	Excessive bleeding	195	55.1
	Abdominal pain	189	53.4
	Severe headache	93	26.3
	Fainting	86	24.3
	Foul smelling vaginal discharge	48	13.6
Advantages of pregnancy and delivery related services(*n* = 368)	For better health care to the women	222	60.3
	Anticipating problems	183	49.7
	For better care to the newborn	178	48.4
	For early detection of health Problems	164	44.6

**Fig. 3:**
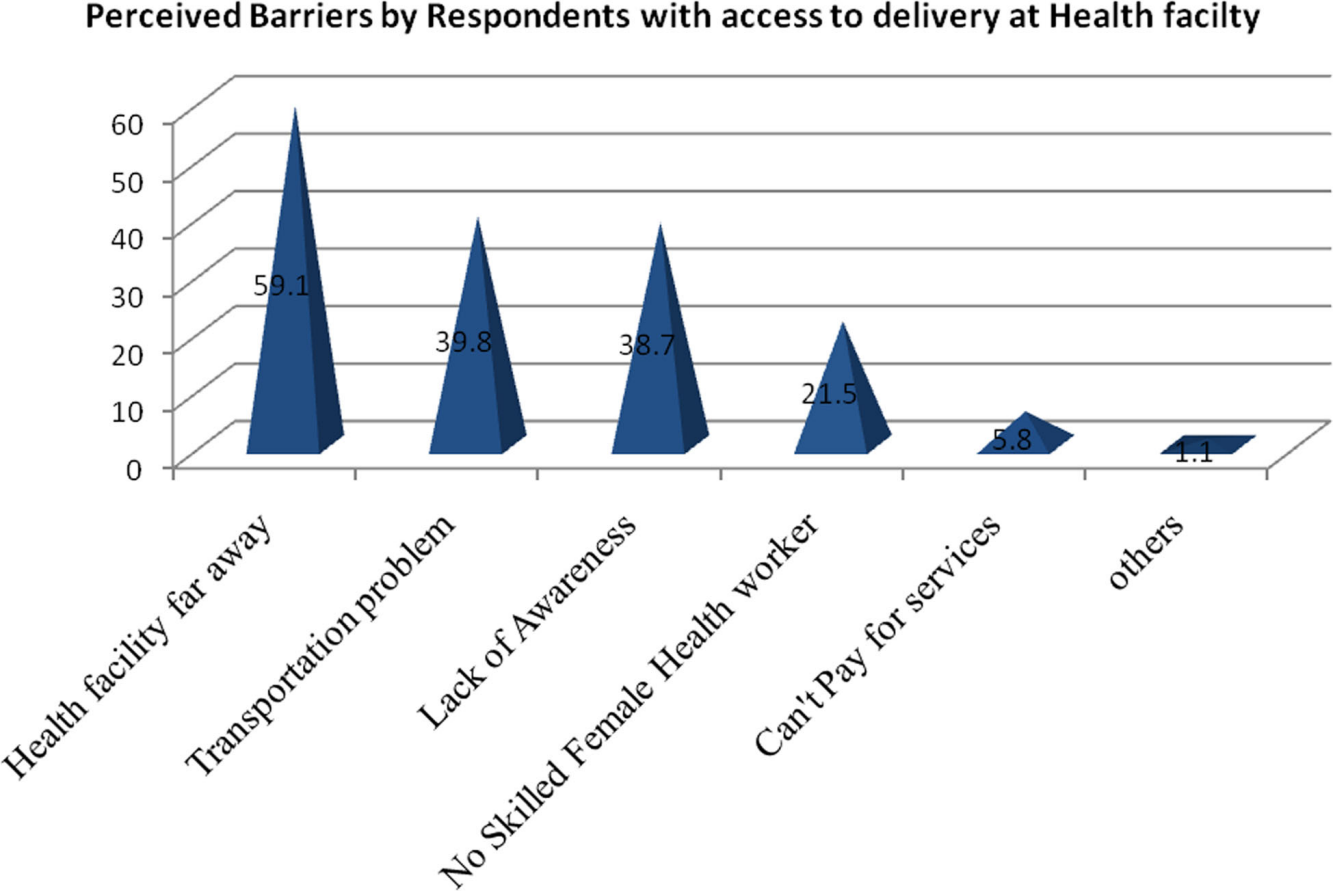
Perceived barriers by respondents with access to delivery care at health facility in Gura Dhamole woreda, Bale zone, Southeast Ethiopia, April, 2017

Three hundred sixty seven (95.8%) of respondents perceived that the ability of delivery attendants during labor and child birth between skilled birth attendant and unskilled birth attendant are not similar (Fig. 3). Among respondents 292 (75.5%) mentioned the availability of HF that can give delivery service in their area and 161(41.6%) rate the easiness of getting institutional delivery service as fair (Table 4).

**Table 4:** Availability of health service, easiness to get service, distance and time taken to reach facility with skilled attendant in Gura Dhamole woreda, Bale zone, Southeast, Ethiopia, April, 2017

Variables	Response	Frequency	Percentage (%)
Presence of HF which gives delivery service in your area	Yes	292	75.5
	No	95	24.5
Rating the easiness for getting institutional delivery services	Very easy	93	24
	Fair	161	41.6
	Very difficult	103	26.6
	Impossible	30	7.8
Distance from nearest HF with SBA in kilo meters	< 5 km	116	30
	5-10 km	126	32.6
	10 km and above	145	37.4
Time taken to nearby HF with SBA	< 1 h	92	23.8
	1-2 h	65	16.8
	2 h and above	230	59.4
Can you get transportation services to visit HF with SBA	Yes	253	65.4
	No	118	30.5
	Do not know	16	4.1
Can you afford to pay for transportation services to visit HF	Yes	211	54.5
	No	176	45.5
Heard about presence of free ambulance service for labouring mother	Yes	348	89.9
	No	39	10.1
Used free ambulance service for transportation to HF during your last pregnancy and child birth	Yes	46	11.9
	No	341	88.1
Decision maker about the past place of delivery	Myself	143	37
	My husband/ Mother- in- law/Others	57	14.7
	Both of us (Me and my husband)	187	48.3

Maternity waiting homes are residential structures built in compounds of HF providing maternity care where mothers far from the institution can stay in the final weeks of their pregnancy. Two hundred eighty seven (74.2%) of respondents have heard about presence of maternity waiting home in nearby HC. Even though most of the respondents have heard about presence of maternity waiting home, only 37(9.6%) of them used it. Those respondents who utilized maternity waiting home 23(59.5%) of the respondents used during labor and child birth, both during pregnancy and child birth 10(27%), after giving birth at home and faced complication during visiting HC 3(8.1%) and others have used it during pregnancy 2(5.4%). The reasons for not using maternity waiting home were far from facility and transportation problem 109(31.1%), lack of information 92(26.3%), closed HC 85(24.3%), not suitable 46(13.1%), preferring home delivery 42(12%) and labor was urgent and sudden 9(2.6%).

### Bivariate and multivariate analysis of factors associated with utilization of skilled birth attendant

Bivariate analysis was done and socio demographic and economic factors residence of respondent, educational status of mother and husband, occupational status of respondents and husband, decision maker on place of delivery, distance and travel time to nearest Health Facility with Emergency Obstetric Care (EmOC) were found significant factors. Depending on the findings from the bivariate analysis, variables were selected for multivariate analysis using binary logistic regression models. The independent variables were recruited based on their level of association that (*p*-value < 0.05) and checking the confounding effect of each variable with other variables. Only those variables that fulfill the criteria on bivariate analysis were entered in multivariate analysis.

In this study, place of residence, educational status of mother, travel time in hours from home to nearest health facility with SBA, decider on place of delivery, ANC visit frequency during last pregnancy, birth preparedness and complication readiness arrangement/action taken ahead of last child birth, knowledge of mothers on obstetric danger signs related to post partum and knowing about presence of maternity waiting home in nearby HC were independent factors associated with utilization of SBA at birth after adjusting the effect of other variables.

Women living in urban areas were about 5 (AOR = 4.74, 95% CI: 1.0–22.36) times more likely to be assisted by skilled birth attendant during child birth compared those residing in rural areas. Women who attended secondary and above educational level were four times more likely (AOR = 4.23, 95%CI: 1.19–14.98) than those mothers with no formal education in utilizing SBA at delivery. Women who were knowledgeable about danger signs of post partum period utilize SBA at birth 2.63 times more likely than that of those who were not knowledgeable (AOR = 2.63, 95%CI:1.19–5.81). More over those mothers who were well prepared (taken ≥2 of the BPACR arrangements/actions) ahead of the last child birth were 4.79 times more likely to deliver in HI with SBA than those mothers who were not well prepared (AOR = 4.79,95%CI:1.85–12.39). Those mothers who had ANC follow up of four and above were about 5.43 times more likely assisted by SBA during delivery as compared to those mother that follow ANC less than four visit (AOR = 5.43,95% CI:2.56–11.55). Those mothers who decide on place delivery jointly with their husbands were about three times more likely to be assisted by SBA at HF during their child birth compared to those mothers who decides only by themselves (AOR = 2.91,95% CI:1.17–7.24). Respondents who have access to a HF within less than 1 h travel were 4.24 times more likely to deliver in a HF with SBA than those mothers who travel more than 2 h (AOR =4.24, 95% CI: 1.18–15.20). In addition respondents who knew presence of maternity waiting home in nearby HC were 3.76 times more likely utilize HF with SBA during delivery compared to those who didn’t knew the availability the waiting home (AOR = 3.76,95 CI%:1.14–12.43) (Table 5).

**Table 5:** Factors associated with utilization of skilled birth attendant at birth in Gura Dhamole woreda, Bale zone, Southeast Ethiopia, April, 2017

Variables	Used Skilled Birth Attendant		COR(95% CI)	AOR(95% CI)
	No Frequency (%)	Yes Frequency (%)		
Socio demographic and economic factors				
Age of respondents				
15–19	43(79.6)	11(20.4)	1	
20–24	87(74.4)	30(25.6)	1.07(0.09,11.76)	–
25–29	86(68.8)	39(31.2)	2.98(0.32,26.86)	–
30–34	15(78.9)	14(21.1)	2.64(0.96,23.54)	–
35–39	33(69.80)	19(30.2)	3.09 (0.24,39.54)	–
Place of residence				
Urban	10(15.4)	55(84.6)	25.03(12.05,52.02)*	4.74(1.0,22.36)*
Rural	264(82)	58(18)	1	1
Family size				
< =4	93(65.5)	49(34.5)	1	
5–6	79(71.8)	31(28.2)	1.77(0.38,8.20)	–
> =7	102(75.6)	33(24.4	1.76(0.61,5.05)	–
Monthly income				
< 500 birr	38(69.1)	17(30.9)	1	
500–100 birr	88(75.2)	29(24.8)	0.26(0.07,1.02)	–
> =1001 birr	147(68.7)	67(31.3)	0.25(0.09,1.10)	–
Educational status of Respondents				
No formal Education	190(77.2)	58(22.8)	1	1
Primary Education	78(72.2)	30(27.8)	1.31(0.78,2.19)	0.61(0.25,1.48)
Secondary and above	6(18.2)	27(81.8)	15.27(6.0,38.83)*	4.23(1.19,14.98)*
Educational status of husband				
No formal Education	162(82.2)	35(17.8)	1	
Primary Education	95(69.3)	42(30.6)	2.84(0.53,15.22)	–
Secondary and above	15(30.6)	34(69.4)	1.75(0.37,8.13)	–
Occupation of the respondent				
House wife	201(71.7)	56 (28.3)	1	
Livestock rearing	66(72.5)	25 (27.5)	0.11(0.16,1.05)	–
Government employed	2(13.3)	13(86.7)	0.38(0.52,2.68)	–
Merchant, Daily labourer and student	5(20.8)	1979.2)	0.17(0.00,4.06)	–
Occupation of the husband				
Farmer	157(71.6)	62(28.3)	1	
Livestock rearing	101(90.1)	11(9.8)	0.29(0.05,1.53)	–
Government employed	5(20)	20(80)	0.12(0.01,1.04)	–
Merchant, Daily labourer and student	9(25.9)	18(74.1)	0.98(0.11,8.69)	–
Service accessibility and utilization factors				
Time taken to nearby health facility with SBA				
< 1 h	28(30.4)	64(69.6)	15.24(8.47,27.41)*	4.24(1.18,15.20)*
1-2 h	46(70.8)	19(29.2)	2.75(1.43,5.32)	2.28(0.91,5.75)
2 h and above	200(87)	30(13%)	1	1
Decision maker about the past place of delivery				
Myself	122(85.3)	21(14.7)	1	1
My husband /Mother- in- l aw/Others	47(82.5)	10(17.5)	1.24(0.54,2.82)	1.07(0.3,3.84)
Both of us (Me and my husband)	105(56.1)	82(43.9)	4.54(2.63,7.83)*	2.91(1.17,7.24)*
ANC Frequency				
< 4 visits	148(83.1)	30(16.9%)	1	1
4 and above	30(27)	81(73%)	13.32(7.50,23.65)*	5.43(2.56,11.55)*
Obstetric factors				
BPACR Status/Arrangement taken				
Not well prepared	156(93.4)	11(6.6)	1	1
Well prepared	118(53.6)	102(46.4)	12.26(6.3,23.87)*	4.79(1.85,12.39)*
knowledge on danger of obstetrics problems related to postpartum				
Not knowledgeable	201(84.5)	37(15.5)	1	1
Knowledgeable	73(49)	76(51)	5.66(3.52,9.1)*	2.63(1.19,5.81)*
Knew about presence of maternity waiting home in nearby HC				
Yes	195(67.9)	92(32.1)	1.78(1.03,3.05)*	3.76(1.14,12.43)*
No	79(79)	21(21)	1	1

## Discussion

In this study 113 (29.2%) of respondents who gave birth were assisted by skilled birth attendant at delivery. This means majority 274 (70.8%) of women gave birth without assistance of SBA at home during the last child birth. The finding of this study agrees with the findings other studies conducted in different parts of Ethiopia such as in Bale zone Goba district (32.9%), southern Ethiopia (Loka Abaya woreda (26.8%) and Cheha district (31%)), national finding of EDHS 2016 (28%) [9, 10, 13, 14] and another study conducted in Bangladesh (30.06%) [15]. However this finding is higher than studies conducted in Arsi zone Munesa woreda (12.3%), Awi zone Akansha Guagusa Woreda (18.7%), northwest Ethiopia Sekela district (12.1%) and a study conducted in northern Nigeria (13%) [12, 16–18]. This difference can be as a result of improvements in accessibility to HIs that provide skilled delivery service and increased interest of utilizing the service due to community mobilization through health development army which has been implemented in the country in recent years. But the finding was lower than studies conducted in western Ethiopia (39.7%), Nepal (48%), Tanzania (44.5%) and Kenya (54 and 40.3%) [19–23]. This difference is as a result of difference in accessing the service as well as due to the fact that women in those countries had better economic and educational status, and the recommended visit of antenatal care service coverage.

In the current study, factors that were significantly associated with SBA utilization at birth by controlling the effect of other variables were place of residence, mother educational status, travel time from home to health facility with SBA, decision maker on the place of delivery, frequency of ANC visit, BPACR arrangement/action taken prior to child birth, knowledge on obstetric danger signs after delivery and knew presence of maternity waiting homes in nearby health center.

In this study pace of residence was independently associated with being assisted by SBA at delivery. Women living in urban areas were about 4.74 times more likely to be assisted by SBA at delivery compared to rural respondents (AOR = 4.74, 95% CI: 1.0, 22.36). This finding agrees with other studies conducted in different part of Ethiopia (12, 18, 25). This is due to the fact that those living in urban area can easily access a HFs that provides skilled delivery service and they don’t need transportation as a HF is near to them, even in case transportation is needed, they can get vehicle than women in rural areas. In rural areas transportation service is not available and majority of the areas had no road and telecommunication infrastructure even to use free ambulance service for laboring mothers.

Those women residing in areas less than 1 h walking distance from home to HF with SBA were about four times more likely to utilize SBA than women walking more than 2 h (AOR = 4.24, 95% CI:1.18,15.20). This finding is similar to other studies conducted in different parts of Oromiya region [13, 16, 24] and other low income countries [20, 23, 25]. Walking is the predominant form of transportation in the study area. Even if transportations like motor cycle are available it is not suitable for laboring women. So women should go on foot or being carried on bed by humans (cultural ambulance) which takes more time to reach HFs. Due to such challenges mothers who were far from HF prefer to give birth at home without assistance of SBA and they go to health facilities after repeated trial of labor at home fails.

Another factor which was statistically related to the use of skilled birth attendants during delivery was women’s education. Women who attended secondary and above education were four times more likely to give birth assisted by skilled birth attendant than those who had no formal education. The finding was also consistent with the finding EDHS of 2016 and other studies conducted in different part of Ethiopia [9, 12, 13, 17, 19] as well as with other studies done in other countries [18, 19, 23]. This is due to the fact that educated women are more likely to develop greater confidence, capabilities to make wise decisions about their own health, aware of difficulties during pregnancy and seek proper health care than those who are not educated. Educated women also easily understand counseling given from health professional and might have more access to written information and they could adapt to modern cultural perspectives like delivery by SBA at health institutions.

In this study woman whose final decision on place of delivery was made by discussing with their partner or husband are more likely to be assisted by SBA at child birth. Women who decide jointly with their husbands on place of delivery were three times more likely to utilize SBA than those decide by only themselves. This finding is consistent with the finding of studies done in central Ethiopia South west Shoa zone and Nigeria [18, 24]. This is as a result of family support in accessing health facilities which improve maternal health services utilization by mothers. If women are encouraged by husbands, they would also get financial and other social supports to go to health facility which will allow them to have health provider assisted delivery [26].

In contrast to this, studies conducted in western Ethiopia have showed that women whom the decision on place of delivery made by themselves were two times more likely gave birth in health institution with SBA compared to mothers whom decision made by others on place of delivery [19]. This might be due to negative effect of too many other people being involved in the decision making process leading to delays in seeking care of SBA attendant during labor and child birth.

Decision on the place of delivery can best made by women who have ANC attendance [16]. The frequency of ANC visit during pregnancy has a positive effect in utilizing skilled attendance. Those mothers who had four antenatal care visits and above were almost five and half more likely to deliver through the assistance of skilled birth attendants than those mothers who had less than four antenatal care visits. This finding is in line with finding of other studies in North West, Western and southeast Ethiopia [12, 27, 28]. This is due to the fact that as the number of antenatal care visits increases, women will be familiar with basic information on pregnancy and delivery related risks that require skilled providers’ assistance.

Another statistically significant factor associated with the use of SBA at birth was Birth preparedness and complication readiness action/arrangement taken ahead of child birth. The finding of this study shows that women who were well prepared (≥2 arrangement or more than average Arrangement) for child birth were about 5 times more likely assisted by SBA at delivery. The finding was consistent with studies conducted in different parts of Oromiya regional state [13, 24, 28] and other study conducted at Amansie West District of Ghana [29]. This is because during ANC in which counseling was given to pregnant women’s on every visit and especially on fourth visit on birth preparedness and complication readiness. Women who were well prepared early were ready to overcome delay that may occur to visit health facility during labor and child birth.

Knowledge of mothers about obstetric danger signs that may occur after delivery was also significantly associated with the utilization of SBA at delivery. Women’s who were knowledgeable (know at least three key danger signs) about obstetric danger signs during post partum were two and half times more assisted by SBA at child birth compared to those who were not knowledgeable. Similarly other studies conducted in other regions of Ethiopia [10, 17] and other countries like Nepal and Ghana were also agrees with this finding [20, 29]. Knowledge of mother on key obstetric danger signs after delivery was very influential factors for mothers to utilize SBA at birth. Because mothers might fear that she may face these obstetric problems after giving birth and to overcome her fear she prefer assistance by SBA during their child birth.

Finally, awareness of mothers about the presence of maternity waiting home in nearby health center was also independently associated with the use of SBA during delivery. Those mothers whom were knew about presence of maternity waiting home in health center was approximately four times more likely assisted by SBA during child birth at health facilities than those not aware of its presence. This is due to the fact that majority of women’s were very far from health institution which give skilled birth delivery and they don’t get transportation service on time, even they don’t get transportation service at all due to poor road expansion in rural areas as well as absence of telecommunication to get free ambulance service. Therefore those pregnant mothers who decide to deliver at health institution prefer to stay at maternity waiting home until the labor start and give child birth.

### Strength of study

As this study is community based study it has reflected the actual situation of the problem in the community that could not be addressed through facility based study.

### Limitation of study

The study cannot allow the establishment of cause and effect relationships as study is cross sectional. There could be recall bias, since the women were asked for events within the last 2 year prior to the survey which increases memory lapse.

## Conclusion

In this study proportion of skilled birth attendant utilization during child birth was still very low. More than two third of women gave birth without assistance of skilled birth attendant at home. Maternal educational status, place of residence, travel time from home to nearest health facility, decision maker on place of delivery, frequency of ANC visit, birth preparedness and complication readiness status ahead of child birth, knowledge on key obstetric danger signs after delivery/during post partum and awareness about presence of maternity waiting home in nearby health facility were factors that are significantly associated with utilization of skilled birth attendant during child birth. It is required to work on strategies that improve the attendance of recommended ANC visits and giving greater attention to birth preparedness and complication readiness as well as awareness creation on obstetric danger signs using different means of behavioral changing communications at facility and community level.

## Data Availability

The questionnaire used for data collection, data and materials used in the study for this manuscript are available at the hand of the author and corresponding authors and can be accessed on demand.

## Abbreviations

ANC: Ante Natal Care
AOR: Adjusted Odds Ratio
BPCR: Brith Preparedness and Complication Readyness
CI: Confidence Interval
COR: Crude Odds Ratio
EDHS: Ethiopian Demographic and Health Survey
EmOC: Emergency Obstetric Care
HEW: Health Extension Workers
SBA: Skilled Birth Attendance
SD: Standard Diviation
SPSS: Statistical Package fore Social Science
